# Primary Fallopian Tube Carcinoma Presenting as a Broad Ligament Fibroid: A Rare Case

**DOI:** 10.7759/cureus.49142

**Published:** 2023-11-20

**Authors:** Jalormy S Joshi, Amardeep Shanoo, Apoorva Dave, Nainita Patel

**Affiliations:** 1 Obstetrics and Gynaecology, Jawaharlal Nehru Medical College, Datta Meghe Institute of Higher Education and Research, Wardha, IND

**Keywords:** pftc, primary fallopian tube carcinoma, diagnostic dilemma, gynecological malignancy, broad ligament fibroid

## Abstract

Primary fallopian tube carcinomas (PFTCs) are quite rare with the incidence ranging from 0.3% to 1.1% amongst all the gynaecological malignancies. Here, we present a rare case of a 44-year-old female (parity-2, live-2 and abortion-2), with one previous classical caesarean section and one vaginal birth after caesarean section (VBAC), bilateral tubal ligation done referred to our gynaecology OPD with complaints of pain in the abdomen since the past six days. The patient also had complaint of spotting per vagina for the past two months. Her ultrasonography and contrast-enhanced CT abdomen and pelvis were suggestive of broad ligament fibroid, which turned out to be a PFTC. Primary fallopian tube malignancies are so rare that this entity may be missed in routine clinical practice and surprisingly noticed during operative procedure or on histopathology reports. Thus, one must be aware of this rare clinical entity and keep it in mind while taking patients on the operating table.

## Introduction

Primary fallopian tube carcinomas (PFTCs) are the rarest form of all gynaecological malignancies. The PFTC incidence rate ranges from 0.3% to 1.1% [1]. The commonest age of occurrence is 40 to 65 years, with the mean age being 55 years [2]. The term was first defined by Reynaud in 1847 [3]. According to population studies, the mean incidence of PFTCs is 3.6 per million women per year [3]. Metastatic carcinoma of the fallopian tube from the ovaries, gastrointestinal tract (GIT), endometrium and breast is more common as compared to primary cancers [4].

Advanced fallopian tube and adnexal carcinomas are mostly misdiagnosed as ovarian carcinomas, so the true incidence could be higher [1]. As shown by histological, molecular and genetic analyses, most of all high-grade serous carcinomas of the ovary and peritoneum have their origin in the fimbrial end of the fallopian tube [5,6]. The staging systems and treatment strategies used for epithelial ovarian carcinomas (EOCs), like cytoreductive surgeries and adjuvant chemotherapy, are also applied to PFTCs [7]. This makes the pre-operative diagnosis of PFTCs extremely difficult [4]. PFTCs have a classical triad of symptoms, which include vaginal bleeding, pelvic pain and vaginal discharge [3]. However, the presence of these symptoms is not sufficient to make a pre-operative diagnosis of PFTCs.

Here, we present a rare case of primary fallopian tube malignancy that we accidentally encountered while operating on a suspected case of broad ligament fibroid based on ultrasound and contrast-enhanced CT scan findings.

## Case presentation

A 44-year-old female, parity-2, live-2 and abortion-2 (P2L2A2), with a previous classical caesarean section and one VBAC, had bilateral tubal ligation done 15 years ago and had been post-menopausal for the past year. She was referred to gynecology OPD with a chief complaint of pain in the abdomen for the past six days. The pain was dull in nature in the lower abdomen, non-radiating, not associated with food intake, and was relieved by medications. The patient also had complaints of spotting per vagina for the past two months, soaking half a pad per day. The patient also complained of difficulty passing urine and constipation. There was a history of decreased appetite and generalized weakness. There were no other gastrointestinal or menstrual symptoms. The patient had no other medical or surgical illnesses. There was no family history of similar complaints or malignancies.

The patient was poorly built, weighing around 42 kg. On examination of the patient, her general condition was moderate; as per the Eastern Cooperative Oncology Group (ECOG) Performance Status, she was grade two; she was afebrile and severely pale, cachexic and malnourished. The patient had tachycardia (pulse rate 122/min) and tachypnea (respiratory rate 22 cycles/min), and her blood pressure was 106/70 mmHg. Her cardiovascular and respiratory examinations were within normal limits.

As per abdominal examination, the abdomen was overdistended and tense with no dilated veins. A vertical infra-umbilical classical caesarean section scar was present until pubic symphysis. An irregular mass of 24 to 26 weeks of uterine size was felt arising from the pelvis with globular enlargement. Bulk of the mass felt towards the left side of the abdomen. The margins of the mass were poorly defined with variegated consistency. Mass was non-mobile in nature. Per speculum examination, the vagina was healthy. The cervix was healthy, effaced and pulled up. On per vaginal examination, the uterus was irregularly enlarged with a 22-24-week size and variable consistency. The mobility of the uterus was decreased; uterine movements were transferred to the cervix. Most of the bulk of the mass was felt on the left side of the abdomen.

**Figure 1:**
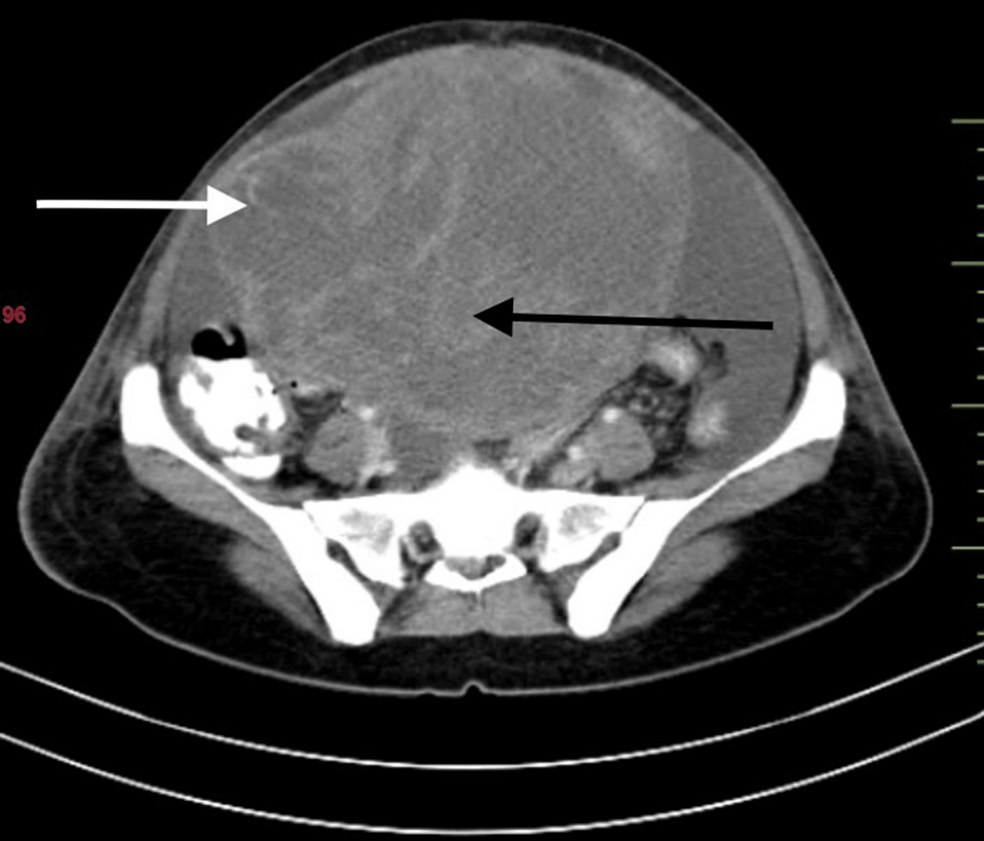
Contrast-enhanced CT abdomen and pelvis axial section showing a solid (black arrow)-cystic (white arrow) mass

**Figure 2:**
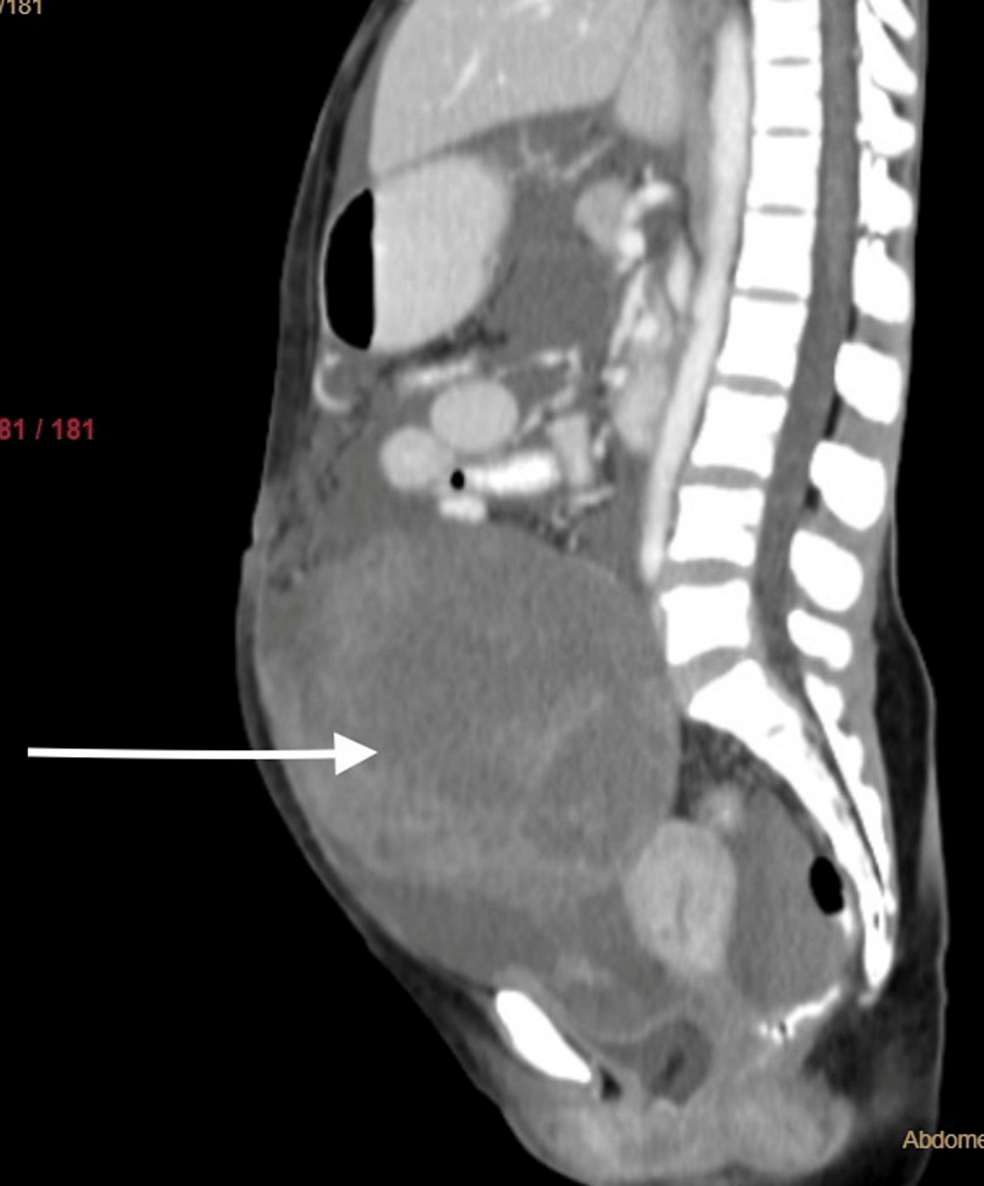
Contrast-enhanced CT abdomen and pelvis sagittal section solid-cystic mass in the right adnexal region (white arrow)

The patient’s hemoglobin (Hb) was 6.3 gm% at the time of admission, total leucocyte count (TLC) was 18,600/cumm and platelets were 2.57L/cumm. CA-125 was 46.9 U/mL. The patient's thyroid-stimulating hormone (TSH) was 6.92 mlU/L, and she was started on tab thyroxine 25 mcg before breakfast. Her renal function test (RFT), liver function test (LFT) and coagulation profile were within normal limits. The patient was transfused with a total of four units of packed red cells (PRCs) to reach Hb levels of 9.6 g/mL. A plain radiograph of the chest (CXR P-A view) was within normal limits. An endometrial biopsy of the patient was taken and sent for histopathological examination, which was suggestive of endometrial tissue and glands in the proliferative phase of the cycle. A cervical smear was taken and sent for cytological examination, which was suggestive of an inflammatory smear with absent malignant cells. Ultrasonography was done, which was suggestive of an ill-defined heterogeneously hyperechoic mass of 15.2x13.8x11.4 cm with free hypoechoic areas within it in the right adnexal region extending superiorly up to the umbilicus. The mass was taking minimal vascularity on Doppler. Endometrial thickness was 6 mm. The right ovary was not visualized, and the left ovary was normal. There was the presence of gross ascites with echogenic debris. Contrast-enhanced CT scan of the abdomen and pelvis was done, which was suggestive of a large, well-defined solid-cystic mass showing heterogenous post-contrast enhancement, measuring 16.3x13.4x13.3 cm, noted in the right adnexal region extending superiorly into the abdomen up to the umbilicus and causing displacement of adjacent bowel loops. The right ovary is not visualized separately. The left ovary appears normal. Omental caking with mesenteric fat stranding is noted. Free fluid in the abdomen and pelvis with bilateral pleural effusion is shown in Figures 1-2.

**Figure 3:**
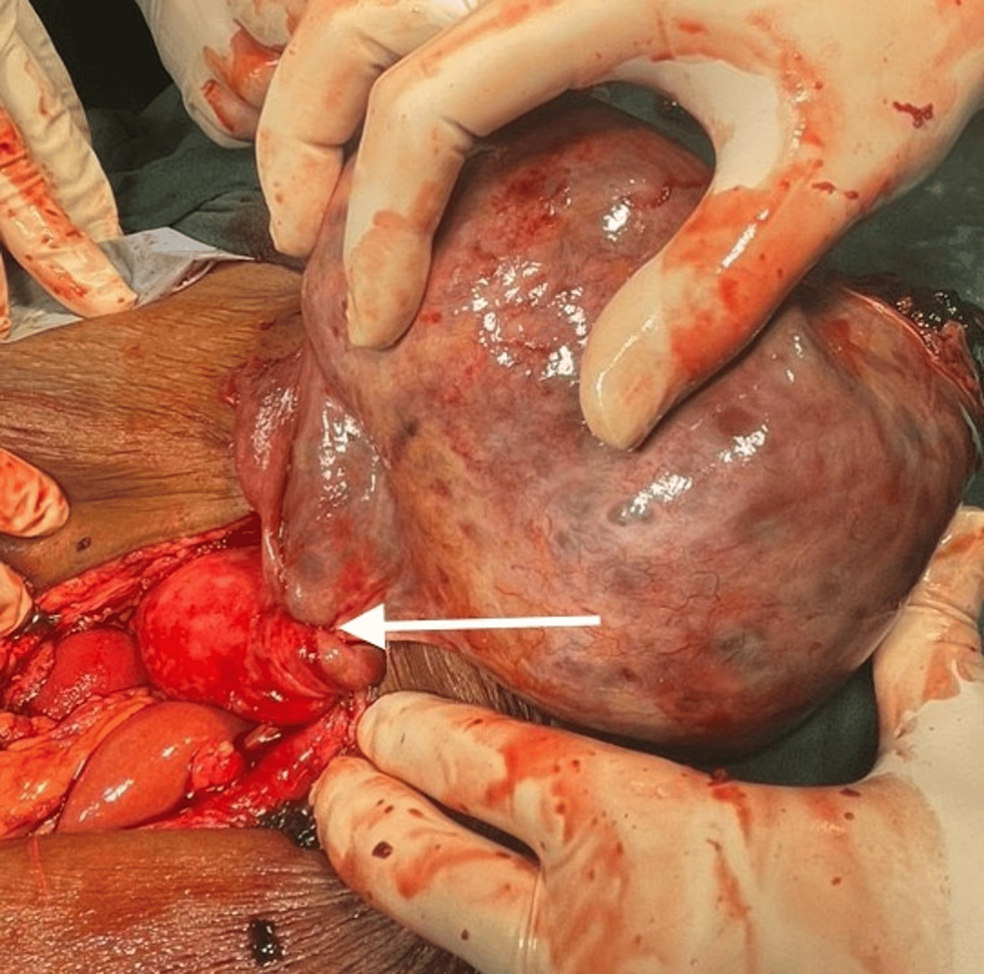
An encapsulated mass arising from right fallopian tube that is twisted three times over the right cornual structures (white arrow)

**Figure 4:**
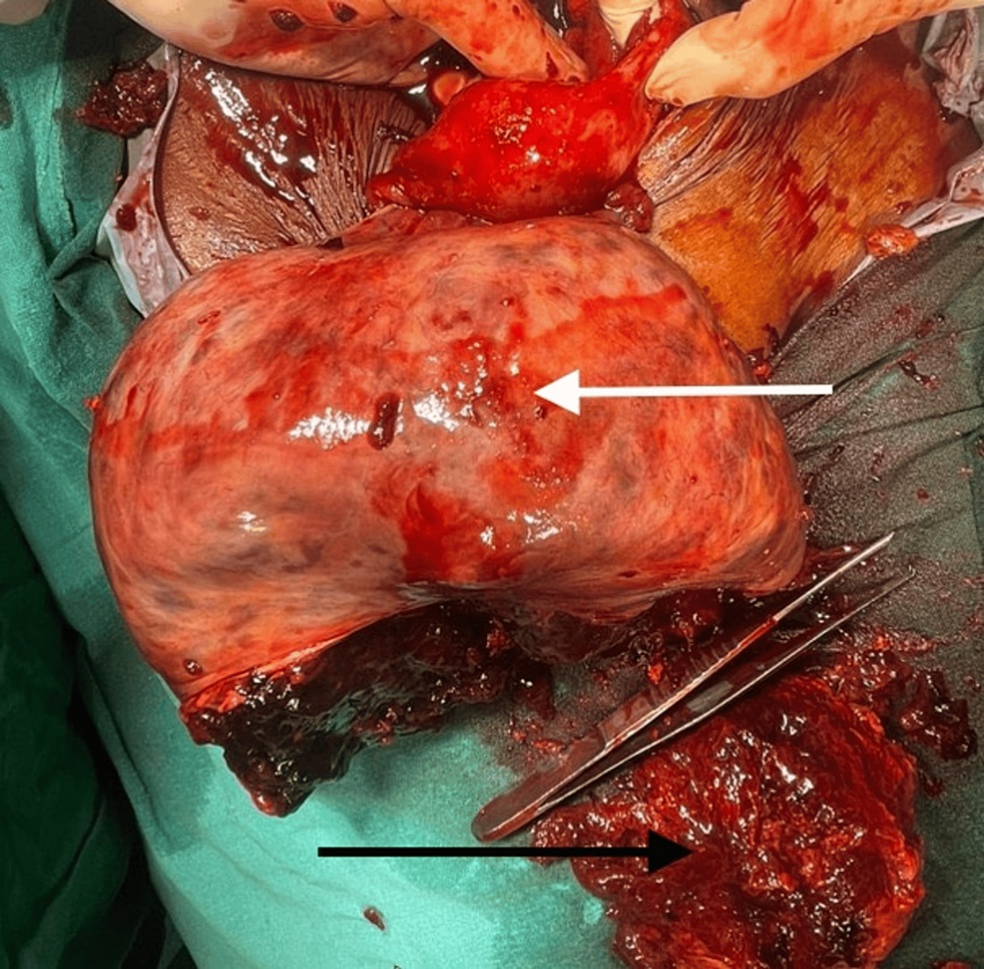
A huge, encapsulated solid-cystic tumour (white arrow) with a broken capsule at the inferior surface and necrotic debris (black arrow)

An exploratory laparotomy was done for the patient. A midline vertical incision was taken adjacent to the scar of the classical C-section. The abdominal cavity was filled with hemorrhagic ascitic fluid. Around four liters of hemorrhagic ascitic fluid were drained from the cavity and sent for cytological examination. A huge, solid-cystic tumour that was encapsulated with a broken capsule at the inferior surface of the tumour was seen, and necrotic debris from the tumour was seen in the peritoneal cavity. The tumour was occupying the lower abdomen, from the pelvis up to the umbilicus, placed in front of the uterus. The right ovary was incorporated into the tumour and could not be identified separately. The tumour was twisted three times over the right cornual structures. The right fallopian tube was visualized along with para-tubal cysts. The left ovary, left fallopian tube, uterus and cervix were visualized and were normal (Figures 3-4).

**Figure 5:**
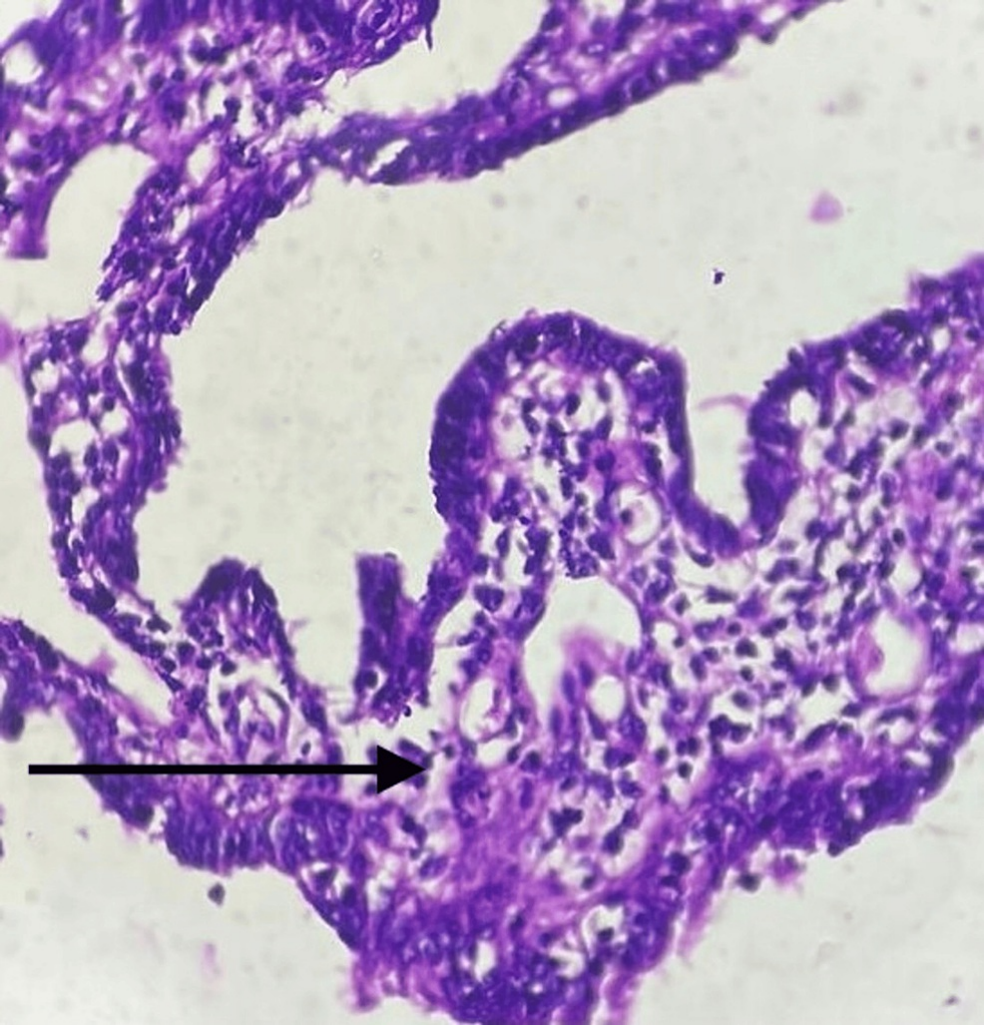
The section shows the lumen filed with fine papillae, tumour cells with a high N:C ratio, loss of polarity and lack of ciliates cells (black arrow)

**Figure 6:**
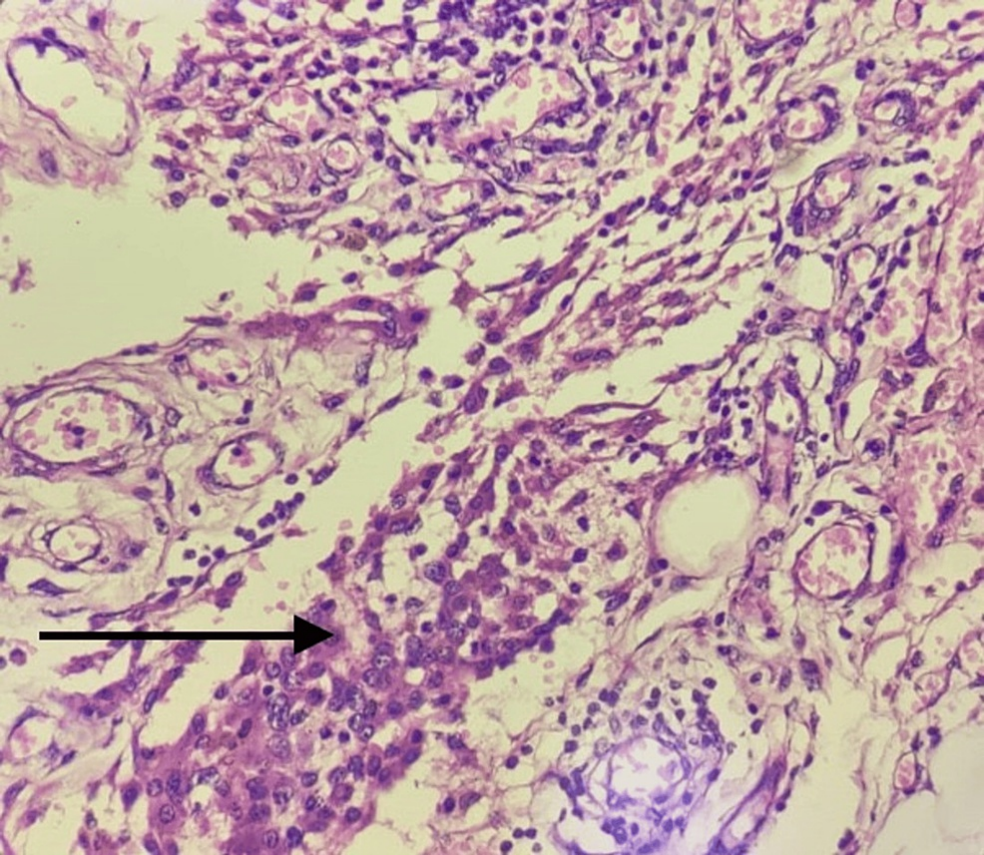
The section shows infiltration of the omental tissue by tumour cells having round to oval nuclei, showing pleomorphism, hyper-chromasia and prominent nucleoli (black arrow)

The tumour mass was excised, along with total abdominal hysterectomy (TAH) with bilateral salpingo-oophorectomy and partial-omentectomy. Lymph nodal dissection was not done as it was not enlarged when palpated intra-operatively. An intraperitoneal drain was placed in situ for five days. The post-operative period was uneventful. Hemorrhagic ascitic fluid cytology was positive for malignant cells. Histopathological examination revealed adenocarcinoma of the fallopian tube, with the right fallopian tube and omental tissue being positive for infiltration by malignant cells, while the right ovary, left ovary, left fallopian tube, uterus, cervix and retroperitoneal nodes were negative for infiltration by malignant cells (Figures 5-6).

By the International Federation of Gynecology and Obstetrics (FIGO) staging of PFTCs, this cancer is staged as FIGO stage 3A. After debulking surgery, on the basis of the histopathology report, paclitaxel 135 mg/m^2 ^IV infusion over three hours with cisplatin 100 mg/m^2^ IP on day 1 with paclitaxel 60 mg/m^2^ IP on day eight, every 21 days for six cycles was given to the patient. The patient tolerated chemotherapy well with minimal gastrointestinal side effects. After that, the patient was lost to follow-up.

## Discussion

Primary malignancies of the fallopian tube are quite rare, and the incidence ranges from 0.3% to 1.1% of all gynaecological cancers. The commonest age of occurrence is 40 to 65 years, with the mean age being 55 years [3]. PFTCs also have associations with tuberculous salpingitis, chronic tubal inflammation, infertility and tubal endometriosis. The high parity of women seems to have a protective effect on PFTCs [3].

The spread is predominantly through the ostia of the tube into the peritoneal cavity. The ovaries and uterus are the most important sites of metastasis. Exfoliation of malignant cells from the primary tumour and their migration towards the fallopian tube to reach the cervix may lead to an early diagnosis of PFTCs by cervical smear examination [3]. Thus, early detection of tumours via cervical cytology may be possible before the disease advances. Initial diagnostic criteria of PFTCs include an essential imaging method in daily practice for the assessment of patients with a potential cancer, such as transvaginal and transabdominal ultrasound [3].

About 90% of fallopian tube carcinomas turn out to be adenocarcinomas by histological examination; among them, half are papillary serous adenocarcinomas [4]. Pelvic and para-aortic lymph node involvement accounts for 34% of all nodal involvements in almost all stages of PFTCs.

Although PFTCs and EOCs share many molecular and clinical traits, PFTCs appear to recur more frequently, and metastasis in distant sites and retroperitoneal nodes is more common [2]. Stage, patient age and, among patients with advanced-stage cancer, residual tumour following initial surgery are crucial prognostic factors for survival [3].

In cases of PFTCs, early metastases, direct peritoneal implantation and lymphatic spread are very common. Due to the advanced stage at the time of diagnosis, the prognosis is mostly poor [4]. Patients with PFTCs typically have low overall survival rates, ranging from 22% to 57%, with a five-year survival rate of 47.4% [7]. However, with early clinical presentation and prompt investigations, an early diagnosis can be made, which in turn improves the prognosis. Preoperative diagnosis is rare, so PFTCs are typically verified by a pathologist [3].

All in all, PFTCs are the rarest among gynaecological malignancies; preoperative diagnosis is difficult in these cases [4]. Total abdominal hysterectomy (TAH) with bilateral salpingo-oophorectomy and pelvic and para-aortic lymph node dissection with omentectomy must be done together for surgical staging. Aggressive cytoreductive surgery should be performed in patients with advanced stages of PFTCs [3].

Primary FTC is a rare and difficult-to-treat disease. FTC is treated similarly to epithelial ovarian cancer because of its clinical behavior, which includes surgical debulking and platinum-based chemotherapy. Although there have been significant improvements in the care and treatment of epithelial ovarian cancer over the past few decades, the survival rate has only slowly increased since the development of platinum-based chemotherapy. However, novel treatments for both primary and recurrent disease, such as immunotherapy, poly (ADP-ribose) polymerase (PARP) inhibitors and anti-vascular endothelial growth factor (VEGF) antibodies, are altering the course of the illness and extending patients' lives [7].

## Conclusions

This case report emphasizes the significance of taking into account unusual and uncommon gynaecological cancers, particularly in patients with alarming clinical characteristics. Early detection and adequate care of primary fallopian tube cancer can dramatically improve patient outcomes. When treating pelvic masses, doctors should always have a high degree of suspicion and be ready to reevaluate their original diagnoses in the event of unexpected findings.
